# Community-level impacts of white-tailed deer on understorey plants in North American forests: a meta-analysis

**DOI:** 10.1093/aobpla/plv119

**Published:** 2015-10-20

**Authors:** Christopher W. Habeck, Alexis K. Schultz

**Affiliations:** 1 Department of Biology, Kutztown University, Kutztown, PA 19530, USA; 2 Department of Education, Kutztown University, Kutztown, PA 19530, USA

**Keywords:** Deer overabundance, forest ecosystem, meta-analysis, *Odocoileus virginianus*, plant diversity, understorey plant community, white-tailed deer

## Abstract

The impacts of introduced or overabundant large herbivores are a concern for the conservation of forest plant communities and the sustainability of ecosystem function. White-tailed deer (*Odocoileus virginianus*) are considered ecologically overabundant in much of North America. Previous work suggests that impacts of deer overabundance are broadly negative and are consequently degrading forests at multiple ecological and taxonomic levels. However, no quantitative synthesis currently exists to verify the generality or magnitude of these impacts. Here, we report the results of a meta-analysis quantifying the effects of deer exclusion on the diversity, cover and abundance of woody, herbaceous and whole community components of forest understories in North America. In addition, we explore the relationships of environmental and experimental factors on the direction and magnitude of plant community outcomes using meta-regression. Using 119 calculated effect sizes sourced from 25 peer-reviewed articles, we constructed 10 community-specific data sets and found strongly positive diversity, cover and abundance responses of the woody community to deer exclusion, but no significant effects for the herbaceous or whole community components of forest understories. Local deer density and time since exclusion were significant moderators of both whole community and woody community richness. Local deer density also moderated the effects of deer exclusion on whole community cover. Plot area, in contrast, showed no relationship to any of the community response outcomes. We suggest that the use of inadequate diversity indices, non-native species replacement or legacy effects of chronic deer overabundance might explain why the herbaceous and whole community components of forest understories showed no diversity or cover responses to deer exclusion. We also suggest some strategies to increase opportunities for future quantitative syntheses of deer impacts on forests, including providing better access to existing and future data. Ultimately, we show that white-tailed deer have strongly negative impacts on forest understorey plant communities in North America, but these impacts are not ubiquitous for all components of the plant community.

## Abstract (precis)

In many regions of the world, exotic or overabundant large herbivores are a concern for the conservation of forest plant communities. Previous work suggests that the impacts of white-tailed deer overabundance in North American forests are broadly negative. However, no quantitative synthesis currently exists to verify the generality or magnitude of these impacts. Using meta-analytical techniques, we show that white-tailed deer have strongly negative impacts on the diversity and abundance of forest understory plant communities, but these impacts are not ubiquitous for all components of the plant community. White-tailed deer have their largest impacts on woody plant species.

## Introduction

At global to regional scales, the composition and functioning of forest ecosystems are being altered by multiple human-assisted stressors (Millennium Ecosystem Assessment 2005; Dornelas *et al*. 2014). Prominent among these stressors are the impacts of large herbivores on plant communities (Putman 1996; Rooney 2001; Vázques 2002; Rooney *et al*. 2004; Tremblay *et al*. 2007). In many regions, the introduction or altered abundance of ungulate herbivores (particularly Cervids) is considered damaging to the sustainability of biodiversity, primary productivity, stand regeneration or economic value of forests (Vázques 2002; Côté *et al*. 2004; Latham *et al*. 2005; Tremblay *et al*. 2007; Apollonio *et al*. 2010; Schumacher and Carson 2013). The ability of ungulate herbivores to alter the composition and diversity of forest plant communities is particularly acute, given that these species occur at high densities in many regions and they are typically selective in their consumption of plant species (Rooney 2001; Apollonio *et al*. 2010; Tanentzap *et al.* 2011; Schumacher and Carson 2013).

The white-tailed deer (*Odocoileus virginianus*) is a native ungulate herbivore in North America that is considered ecologically overabundant in much of its native range, meaning that this species occurs at densities that negatively impact biological diversity, productivity and/or the functioning of ecosystems (McShea *et al.* 1997; Rooney 2001; Côté *et al.* 2004; Carson *et al.* 2014). An accurate estimate of white-tailed deer density and their ecological impacts prior to European settlement are unknown in North America. McCabe and McCabe's (1997) pre-settlement estimate of 3.1–4.2 deer km^−2^ for North America is commonly cited, but relies on some assumptions that are hard if not impossible to verify (e.g. hunting pressure from Native Americans and natural predators). Conversely, empirical estimates of current deer density are widely available at small spatial scales (e.g. states, counties and parks) and typically exceed McCabe and McCabe's (1997) continent-scale pre-settlement estimate (Russell *et al.* 2001; Côté *et al.* 2004), particularly in eastern North America (Abrams and Johnson 2012; Bressette *et al.* 2012; Begley-Miller *et al.* 2014). Observational and experimental studies conducted over the past several decades indicate that contemporary white-tailed deer populations can affect the diversity and functioning of forests at levels of biological organization from species (Eschtruth and Battles 2009; Waller and Maas 2013; Thomas-Van Gundy *et al.* 2014) to communities and ecosystems (Ritchie *et al.* 1998; Bressette and Beck 2013; Murray *et al.* 2013), and across taxonomic groups such as plants (Rooney 2009; Dornbush and Hahn 2013), vertebrates (deCalesta 1994; McShea and Rappole 1997) and invertebrate organisms (Ruhren and Handel 2003; Takada *et al.* 2008; Dávalos *et al.* 2015). The magnitude of these effects, however, are neither equal across levels of biological organization or geographical location, nor are they always negative (Royo *et al.* 2010*a*) nor do they always exist (Kraft *et al.* 2004; Collard *et al.* 2010; Aronson and Handel 2011; Levine *et al.* 2012).

Although the prevailing view is that white-tailed deer have broadly negative impacts on understorey plant communities in forests (Rooney 2001; Côté et al. 2004; Carson et al. 2014), there has been no systematic and quantitative synthesis to validate this widely held view (although see Gill and Beardall 2001 for European deer). Also lacking is an understanding of how environmental or experimental contexts influence the direction and magnitude of species, community or ecosystem-level responses to white-tailed deer herbivory. Certainly, there are inherent complications with synthesizing research related to this particular topic, and these complications probably limited the potential for quantitative synthesis in the past. For instance, the focus of many experiments is limited to specific management scenarios (e.g. clear-cuts, mature forest preserves), species or functional groups of plant communities (e.g. *Trillium* spp., Liliaceae, tree species) or land-use histories (e.g. logged vs. post-agricultural). Also, variation in local deer densities and the length of experiments hinder generalization across a limited number of studies. However, new opportunities for quantitative synthesis have emerged due to an ever-growing number of experiments conducted and published during the past several decades. Here, we endeavoured to synthesize a portion of this work with a specific focus on community-level indices for understorey plants.

Our goals were 3-fold: (i) to quantify the ecological effects of white-tailed deer in North American forests, specifically for community-level measures of understorey plants, (ii) to understand which, if any, environmental or experimental factors potentially influence the direction and magnitude of these effects and (iii) to suggest experimental and reporting strategies for new or existing studies, specifically to strengthen opportunities for similar synthesis projects in the future. We employ a systematic meta-analytical and meta-regression approach to realize these goals, and limit our analysis to peer-reviewed experiments that use barriers (e.g. fences) to exclude white-tailed deer as a treatment factor. We take this ‘all or nothing’ approach to deer density rather than a gradient approach, not because it is the best option, but because there are currently too few published studies that distribute research plots along a deer density gradient (Horsley *et al*. 2003; Nuttle *et al*. 2014) to perform a robust quantitative synthesis.

We predict that the community-level effects of deer exclusion on understorey plants (i) will be generally positive, but will differ among plant functional groups and community indices of diversity, cover and abundance and (ii) will increase in magnitude with estimates of local deer density, time since the initiation of deer exclusion and experimental plot area.

## Methods

### Data collection

All peer-reviewed articles that met the following criteria were included in this meta-analysis:

1. Experiments were conducted within the native range of white-tailed deer in forest ecosystems. However, we excluded experiments that occurred in timber plantations because these sites might have unnaturally depauperate understories due to herbicide treatments or other artefacts of timber management (e.g. disking, mechanical shrub removal, monotypic canopy structure and composition).
2. The response of understorey plants to deer exclusion was reported for the whole community, and/or separately for the woody and herbaceous components of the community. The defining criteria and sampling procedures for understorey flora varied somewhat among authors and/or experiments. Here, we accepted any data the authors considered to be derived from forest understorey plant communities. We included the following community response variables: species richness, Shannon *H* diversity, per cent cover and abundance (e.g. direct counts of individuals).
3. Articles reported the area (i.e. size) of the exclusion and control plots, and indicated the time since the exclusion treatments had been established. In a few experiments, plot area and/or year of establishment varied. In those cases, we computed and used the average value for analysis.
4. Replication of treatments was unambiguously reported. Means and variances were reported in text, table or graphical form, or were made available to us by the author(s).

On 15 April 2014, we collected candidate articles by searching the ISI Web of Knowledge database using the following search string: TS = ((‘white-tailed deer’ OR ‘Odocoileus virginianus’) AND (exclu* OR fenc* OR barrier)).

### Data extraction and classification

**Figure 1.**
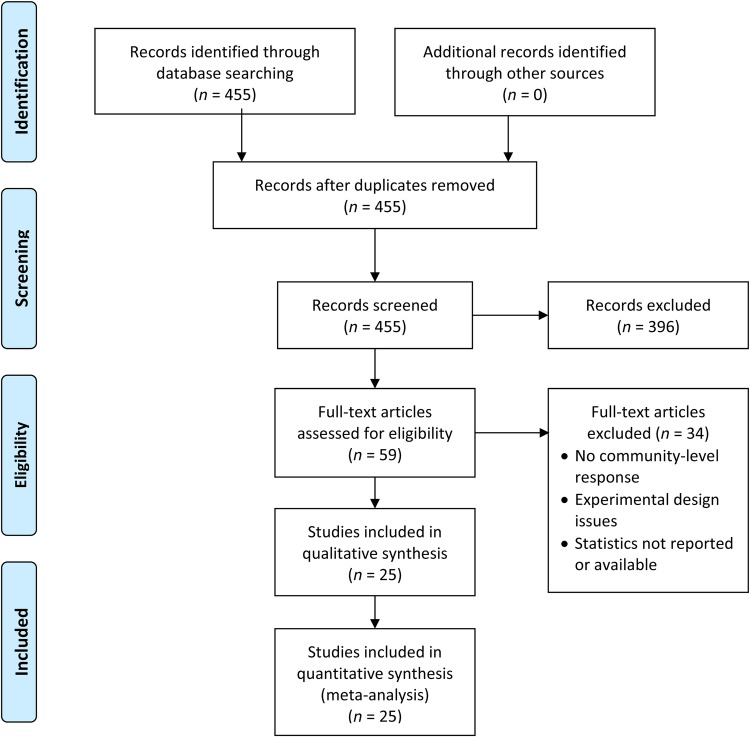
The iterative screening process used to exclude or retain articles for this meta-analysis, formatted as a PRISMA flow diagram (Moher *et al.* 2009).

We used a systematic process to exclude articles that did not meet the inclusion criteria (Fig. 1). Initially, we scanned the title to exclude articles that obviously did not meet our criteria (e.g. title indicated that the experiment focussed on a species other than white-tailed deer, or was conducted outside of North America). We then read the abstracts of all articles remaining after the title screening, and finally, we read the methods and results sections of all articles that remained after the abstract screening. If an article met all of our inclusion criteria, we extracted the means, variances and sample sizes from the appropriate text, tables and/or figures. When necessary, we used the Java program Plot Digitizer (version 2.6.2; Huwaldt 2012) to extract data from scanned figures. When some or all response statistics of interest were not reported, but likely existed, we attempted to procure them by contacting the author(s). If an article reported response outcomes for more than one time period, we extracted the data for only the longest time period reported. However, if an article reported response outcomes from multiple sites or conditions, we extracted these data as separate outcomes and dealt with their non-independence statistically (description to follow).

In addition to the community response variables of interest, we extracted additional information (if available) from each article to use as potential explanatory variables of effect size direction and magnitude. These variables included but were not limited to location name, geographic coordinates, forest type (as reported by authors), deer density and plot size. Finally, raw data from articles were grouped into separate data sets based on the component of the plant community sampled (whole, woody and/or herbaceous) and the response outcome index reported (richness, Shannon *H* diversity, per cent cover and abundance).

### Meta-analysis

We performed all calculations and statistical analyses using functions available in the metafor package (version 1.9-6; Viechtbauer 2010) within the R open-source software environment (version 3.2.0; R Core Team 2015). All data, R code and output used in this meta-analysis are available as digital appendices **[see** **Supporting Information****]**. We used the means, standard deviations and sample sizes of control and deer exclusion plots from each outcome to calculate Hedge's *g* effect sizes (Hedges 1981) and associated sampling variances using the default parameters in the escalc() function. Hedge's *g* is the standardized mean difference between treatments. We chose to use Hedge's *g* over other effect sizes because of its common use in ecology literature for comparing two means (Nakagawa and Santos 2012; Rosenberg *et al*. 2013) and it includes a correction for small sample sizes, which was an issue with our data (Rosenberg *et al*. 2013). The value of Hedge's *g* is >0 when deer exclusion barriers result in an increase in a given community response outcome and <0 if the opposite occurs.

Using restricted maximum likelihood estimation in the function rma.mv(), we ran multi-level random-effects (MLRE) models without moderators for each data set that had five or more effect sizes derived from three or more articles. We took this conservative approach in our decision to analyse or not analyse a data set in an attempt to reduce the potential for false interpretations based on small sample sizes. Many factors (e.g. sampling methodology, soil type and climate) are more likely to be similar within than across experiments and locations. Multi-level random-effects-type meta-analytical models are specifically designed to account for non-independence among effect sizes by allowing for the addition of a random term in the model (Viechtbauer 2010; Nakagawa and Santos 2012). We included ‘locale’ as a random term in each model to account for the potential lack of independence among effect sizes derived from the same article and/or the same experimental infrastructure. The general effect of deer exclusion on community response outcomes was considered significant when the 95% confidence intervals (CIs) for the overall mean effect calculated by the model did not bracket zero. In the absence of an accepted numerical benchmark in ecology (Lortie *et al*. 2015), we interpret the magnitude of the Hedge's *g* effect sizes as suggested by Cohen (1988): small ≥0.20, medium ≥0.50 and large ≥0.80. Heterogeneity among effect sizes within data sets was assessed using the *Q*-statistic. Large *Q* values suggest that differences between effect sizes within a data set do not estimate a common population mean, and thus could vary for reasons other than sampling error (e.g. due to some environmental factor; Hedges and Olkin 1985). We tested for the significance of *Q* using a χ^2^ distribution.

In an attempt to explain residual heterogeneity and understand the potential effect of contextual factors on the response of plant communities to deer exclusion, we ran separate MLRE models that included a single moderator (i.e. meta-regression), given the criteria that the data set had ≥10 effect sizes calculated from five or more articles. Again, we took this conservative approach to ensure a minimal level of power for the analyses. The moderators included in these meta-regression models were deer density (per km^2^), plot size (m^2^) and time since plot establishment (years). To guide inference, we used Akaike's information criteria for small sample size (AIC_c_) to indicate whether meta-regression models were more or less parsimonious (ΔAIC_c_ > 2) than the null (intercept only) model.

We tested for publication bias using Egger's regression test (Egger *et al*. 1997; Sterne and Egger 2005) by modifying the MLRE models to include the standard error of the effect sizes as a moderator. When the intercept of this regression test significantly deviates from zero, the overall relationship between the precision and size of studies included in the data set is considered asymmetrical, and therefore, biased (Sterne and Egger 2005). We considered analyses to be biased if the intercept differed from zero at *P* = 0.10 (as in Egger *et al*. 1997).

The sensitivity of meta-analytical studies is also vulnerable to outliers and influential data points. However, diagnostic tests for identifying, and rules for excluding, these types of cases are still evolving, particularly for multivariate/multi-level meta-analytical models (Viechtbauer and Cheung 2010). We evaluated the sensitivity of our analyses by comparing fitted models with and without effect sizes that we defined as influential outliers. In lieu of other options currently available within the package metafor when using the rma.mv() function, we define influential outliers as effect sizes with hat values (i.e. diagonal elements of the hat matrix) greater than two times the average hat value (i.e. influential) and standardized residual values exceeding 3.0 (i.e. outliers; Stevens 1984; Viechtbauer and Cheung 2010; Aguinis *et al*. 2013).

## Results

### Database, data sets and diagnostics

We retrieved 455 articles from our database search, 25 of which remained suitable for inclusion in our meta-analysis after our iterative screening process. We excluded articles for a variety of reasons, but primarily because they did not employ deer exclusion barriers in their experimental design or their focus was not on white-tailed deer, North American forests or community-level responses of understorey plants. From the 25 articles that were retained, we calculated 119 effect sizes, which were partitioned into 10 separate data sets (Table 1). The experiments described in the articles were conducted in 13 states and 1 Canadian province. The articles were published between 2000 and 2014 in 16 different journals.

**Table 1.** Characteristics of the data sets in the meta-analysis, including the number of articles and outcomes derived from those articles and the mean (range) of experiment-specific factors used in meta-regression models.

Community index	Articles	Outcomes	Deer (km^−2^)	Plot (m^2^)	Years since exclusion
Whole richness	6	14	43 (8–100)	455 (4–4000)	11 (2–18)
Whole diversity	3	7	42 (14–100)	801 (144–4000)	11 (7–16)
Whole cover	6	23	17 (13–37)	442 (4–4000)	8 (2–16)
Woody richness	11	19	29 (5–82)	2255 (4–40 000)	10 (3–18)
Woody diversity	3	5	25 (5–37)	95 (18–144)	5 (3–7)
Woody cover	5	8	10 (5–23)	222 (4–400)	6 (3–13)
Woody abundance	6	7	26 (12–44.5)	510 (100–2500)	11 (4–18)
Herbaceous richness	7	15	33 (5–82)	160 (4–400)	9 (3–18)
Herbaceous diversity	4	7	24 (5–37)	91 (2–180)	5 (2–8)
Herbaceous cover	8	14	25 (5–67)	144 (4–400)	5 (3–13)

We detected publication bias in the data sets evaluating whole community cover (*P* = 0.010), whole community richness (*P* = 0.005), woody community richness (*P* = 0.001), woody community diversity (*P* < 0.001) and herbaceous community diversity (*P* = 0.022). We did not detect influential outliers in any of the data sets. A significant amount of residual heterogeneity remained for all models (*P* = <0.001) except those evaluating woody community cover [*Q* = 12.2, degrees of freedom (df) = 7, *P* = 0.095] and woody community abundance (*Q* = 6.5, df = 6, *P* = 0.371). Five of the 10 data sets had a sufficient number of articles and outcomes (i.e. ≥5 and ≥10, respectively) to explore the potential relationship between environmental moderators and plant community response outcomes: whole richness, whole cover, woody richness, herbaceous richness and herbaceous cover.

### Meta-analysis

**Figure 2.**
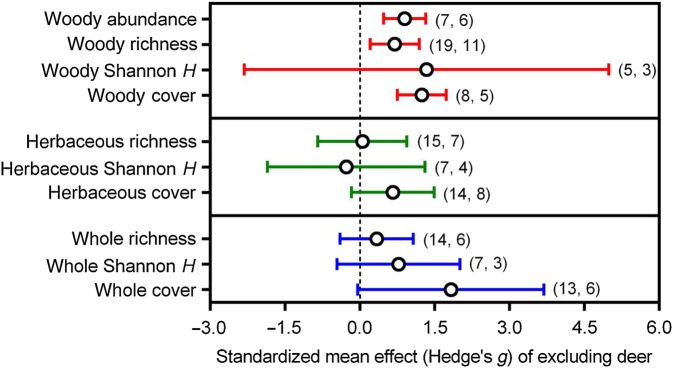
Standardized mean Hedge's *g* effect sizes ± 95% CIs for the community-level response of forest understorey plant groups to white-tailed deer exclusion. The numbers in parentheses (*a*, *b*) represent *a* the number of effect sizes used in the models and *b* the number of independent articles those effect sizes were sourced from.

The overall effect of white-tailed deer exclusion on understorey species richness was not significant for the whole or herbaceous community, but strongly positive for the woody community. There was no evidence that species diversity is influenced by white-tailed deer exclusion for any component of the plant community. Excluding white-tailed deer did not influence whole or herbaceous community cover, but had a strongly positive effect on woody community cover. Finally, the overall effect of white-tailed deer exclusion on woody community abundance was strongly positive (Fig. 2).

### Meta-regression

The community-level effects of deer exclusion on forest understorey plants were not related to the size of experimental plots. The effect of deer exclusion on whole and woody community species richness increased with local deer density (Table 2; whole ΔAIC_c_ = 1.24, woody ΔAIC_c_ = 12.17), whereas the effect of deer exclusion on whole community plant cover declined with local deer density (Table 2, ΔAIC_c_ = 97.60). The influence of deer exclusion on whole community richness increased as the years since exclusion increased (Table 2, ΔAIC_c_ = 7.77), whereas the effect size for woody community richness declined with the length of exclusion (Table 2, ΔAIC_c_ = −2.57).

**Table 2:** Slope (*β)*, 95% CIs and *P*-values for multi-level meta-regression models exploring relationships between deer exclusion effects and experiment-specific factors. The sign of the slope indicates the magnitude and direction ([+] increasing or [-] decreasing) by which the community index changes in relation to each unit increase in the experiment-specific factor. Slopes with *P* < 0.05 are bolded.

Community index	Deer (km^−2^)		Plot area (m^2^)		Years since exclusion	
	*β* (±*CI*)	*P*	*β* (±*CI*)	*P*	*β* (±*CI*)	*P*
Whole richness	**0.025 (0.014, 0.035)**	<0.001	0.001 (−0.001, 0.001)	0.062	**0.158 (0.09, 0.227)**	<0.001
Whole cover	**−1.358 (−1.623, −1.093)**	<0.001	0.001 (−0.001, 0.002)	0.485	0.062 (−0.095, 0.219)	0.438
Woody richness	**0.027 (0.011, 0.043)**	0.001	0 (−0.0001, 0)	0.351	**−0.697 (−1.014, −0.379)**	<0.001
Herbaceous richness	0.024 (−0.012, 0.06)	0.188	−0.001 (−0.007, −0.006)	0.881	0.019 (−0.119, 0.157)	0.793
Herbaceous cover	0.027 (−0.017, 0.071)	0.229	−0.001 (−0.008, 0.006)	0.757	0.003 (−0.267, 0.273)	0.983

## Discussion

Our meta-analysis confirms the widely held belief that reducing the density of white-tailed deer—to zero in this case—has positive impacts on understorey plant communities in North American forests (McShea *et al.* 1997; Côté *et al.* 2004; Carson *et al.* 2014). In addition, we found that local environmental and experimental factors can partially explain the magnitude and direction of some of these impacts. However, the impacts of deer exclusion were not ubiquitous for all components of the forest understorey. Generally, the woody community responded positively to deer exclusion, whereas the herbaceous and whole community components were insensitive to the effects of deer removal. Further, we found no overall impact of white-tailed deer exclusion on Shannon *H* diversity for any component of the plant community. Although not an exhaustive list, we suggest three non-exclusive hypotheses to explain why the herbaceous understorey community, in particular, did not exhibit positive responses to deer removal: (i) the plant community indices reported in the literature are insensitive to the types of negative impacts that white-tailed deer have on herbaceous understorey communities, (ii) native herbs are being replaced by non-native herbs and (iii) chronic deer overabundance has degraded the forest understorey community so severely that recovery of herbaceous species is not probable in the short term (if ever).

Regarding the first hypothesis, it would seem from this meta-analysis that the general effects of white-tailed deer removal do not influence herbaceous understorey communities in North American forests, at least for the indices explored in our analysis (i.e. species richness, Shannon *H* diversity and per cent cover). Nonetheless, published experiments of white-tailed deer-mediated shifts in the composition and dominance of understorey herb species are common (Balgooyen and Waller 1995; Rooney 2001; Royo *et al.* 2010*b*; Goetsch *et al*. 2011; Abrams and Johnson 2012; Begley-Miller *et al*. 2014). The lack of herbaceous response in this analysis, therefore, could be partly or entirely due to shortcomings of the indices commonly chosen to characterize communities (Frerker *et al*. 2014), rather than an indication that deer are ecologically benign vis-à-vis the herbaceous community. Community indices that lack information about species identity can fail to identify compositional shifts of potentially great ecological importance (e.g. McLachlan and Bazely 2001; Parody *et al*. 2001; Avolio *et al*. 2014). Species richness and diversity measures are easy to calculate and useful in many contexts (Gotelli and Colwell 2001; Magurran 2013), including for woody species in this meta-analysis. However, these traditional indices are probably most useful when they are evaluated in combination with other indices that provide information about differences in species identity among treatments or plots, particularly because species identity often dictates the functional consequences of diversity (Díaz *et al.* 2003; Olden *et al*. 2004). Currently, indices that account for changes in composition are rarely included in published reports of deer impacts on plant communities (although see Kain *et al.* 2011; Tanentzap *et al*. 2011). We suggest that new and existing data sets be (re)analysed to include composition-specific indices of community diversity (e.g. measures of species or functional group (dis)similarity; Anderson *et al*. 2011) along with formal tests that indicate compositional differences among treatment levels (e.g. permutational multivariate analysis of variance; Anderson 2001) such that potentially important effects of deer on plant communities are not masked or misconstrued by the use of inadequate (e.g. univariate) community diversity measures.

Continuing with the idea that ‘species identity matters’, our second hypothesis posits that deer simultaneously suppress native plants and facilitate the establishment and proliferation of non-native species, and the consequences of these synchronous interactions—species replacement and/or the alteration of species dominance—are the underlying causes for the non-significant effects of deer removal on herbaceous communities. Several recent experiments have shown that non-native herbs are more abundant in areas accessible to white-tailed deer (Eschtruth and Battles 2009; Knight *et al.* 2009; Abrams and Johnson 2012; Dornbush and Hahn 2013; Kalisz *et al.* 2014), the mechanisms of which are likely both consumptive and non-consumptive. Evidence for consumptive effects stem from the observation that relatively fewer non-native compared with native forbs are consumed by deer, which can facilitate the dominance of the former (Eschtruth and Battles 2009; Kalisz *et al.* 2014). Non-consumptive effects are associated with, among other factors, deer-mediated secondary dispersal of non-native propagules (Myers *et al.* 2004), and disturbances to the forest floor that tend to favour the establishment of non-native plants (Knight *et al.* 2009; Heckel *et al.* 2010; Chips *et al.* 2014; Dávalos *et al.* 2015). Regardless of the mechanism(s) driving the suppression of native plants and proliferation of non-native ones, the result could manifest as similar levels of diversity and abundance inside and outside of deer exclosure plots, particularly if richness and evenness are varying in opposite directions. However, we know of no study—including those included in this meta-analysis—that successfully links invariant herbaceous diversity or abundance values to strong differences in species provenance between deer access and exclosure plots. Given the growing understanding that the sustained degradation of many forest ecosystems is a combined function of overabundant herbivores and invasive plants (Knight *et al.* 2009; Heckel *et al.* 2010; Kalisz *et al.* 2014), future experiments should continue to address how white-tailed deer influence the distribution, abundance and ecological impacts of non-native plants in North American forests.

Finally, a nonresponsive herbaceous community after deer removal could indicate a legacy effect of chronic deer overabundance. Researchers rarely report or have information on the long-term patterns of deer density for their experimental sites. However, Côté *et al.* (2004) indicate that widespread concerns related to deer overabundance in the USA escalated rapidly beginning in the mid-20th century, and these concerns continue today. It is probable, therefore, that deleterious effects of deer overabundance have been sustained over a great portion of their native range for multiple decades. The impacts of chronic deer overabundance are thought to limit the recovery rate of plant communities experiencing reductions or removal of deer (Carson *et al*. 2014; Royo *et al*. 2010*b*; Nuttle *et al*. 2014). Tanentzap *et al.* (2012) outlined several mechanisms whereby a history of chronic deer overabundance can slow or stop the recovery of forest understories. We introduce the authors' ideas here, but readers should refer to the original article for a detailed description of the following mechanistic hypotheses, particularly because we describe them strictly within the context of deer removal rather than deer reduction treatments. Firstly, Tanentzap *et al.* (2012) suggest that some deer-preferred herbaceous species (e.g. *Trillium grandiflorum*) have such low levels of energy reserves remaining for replacement of stems and leaves in heavily impacted areas that they are not visually identified in the system even decades after the removal of deer herbivory (Koh *et al*. 2010). Secondly, herbaceous recovery may be limited because no local or regional sources of propagules are available to allow for the re-establishment of species lost due to the pressures associated with long-term deer overabundance (McLachlan and Bazely 2001). Finally, the mechanistic explanation, termed the recalcitrant understorey hypothesis, suggests that recovery does not occur because the majority of understorey plants are competitively excluded by one or a few species that became dominant specifically because they are unpalatable to white-tailed deer (e.g. ferns and ericaceous shrubs; Royo and Carson 2006; Goetsch *et al.* 2011; Frerker *et al.* 2014; Nuttle *et al.* 2014). Of the hypotheses described above, the recalcitrant understorey hypothesis currently has the most empirical support (Royo and Carson 2008; Royo *et al*. 2010*b*).

Above, we focussed on the herbaceous community in our attempt to explain why we found no overall response to deer removal for the herbaceous and whole components of forest understories. We took this approach because we assume that the lack of response by the whole community is driven by the herb community, particularly in light of the strong positive effects deer removal had on woody plants. We suggest two potential reasons why the woody community exhibited a strong response to deer removal when the herbaceous community did not: (i) the re-establishment of tree species is more likely to occur over shorter timescales because, even after decades of deer overabundance, there is a relatively constant local rain of propagules sourced from mature individuals in the canopy, whereas re-establishment of locally extirpated herbaceous species depends more strongly on long-distance seed dispersal, and (ii) a variety of vertebrates (e.g. birds and squirrels) are effective long-distance dispersers of tree propagules over short timescales, thus speeding up the process of woody species re-establishment. In contrast, the re-establishment of many herbaceous plants is probably more dispersal limited than trees due to slow clonal reproduction (Whigham 2004) or a general lack of long-distance secondary dispersal mechanisms (Handel *et al*. 1981; Cain *et al*. 1998; Kalisz *et al.* 1999).

Using a meta-regression approach, we found some evidence that environmental and experimental factors can potentially influence the direction and magnitude of deer exclusion effects on understorey communities. However, we suggest readers take caution when drawing inferences from these models for two reasons: (i) because moderators generally exhibited wide gaps across their value gradient and (ii) although we already took a purposefully conservative approach, most of these models are based on a limited number of articles and outcomes. That said, it seems that the choice of plot size when conducting deer exclusion experiments has little effect on plant community response; none of the community indices evaluated here were related to plot size. Somewhat surprisingly, the influence of deer exclusion on whole cover showed a strongly negative relationship with deer density, which ranged from 13 to 37 deer km^−2^. We posit that this trend could be related to the aforementioned legacy effects of high deer density (Royo and Carson 2006; Goetsch *et al.* 2011; Frerker *et al.* 2014; Nuttle *et al.* 2014). In other words, chronic overabundance of deer may have created a situation where recovery is less probable or takes more time than in areas with historically lower deer densities. Local deer density also mediated the effects of deer removal on whole and woody community richness, but the slopes of these relationships were modest. In contrast, time since deer exclusion emerged as a strong moderator of both whole and woody species richness. An increasing effect of deer exclusion for whole plant richness over time may indicate a delayed benefit of exclusion for herbaceous species, i.e. recovery of the herbaceous community takes a long time (Handel *et al*. 1981; Cain *et al*. 1998). However, the model evaluating herbaceous richness over time since exclusion does not lend support to this hypothesis. Regarding woody species richness, the decline in effect size over time might indicate losses due to competition among species as a post-deer removal (i.e. post-disturbance) explosion of seedlings either mature into saplings or die for lack of resources. In other words, tree species richness would be expected to gradually decline during the stem exclusion phase of forest regeneration (Kozlowski 2002) after the pressure of deer herbivory is removed. To our knowledge, however, this scenario has not been explored. Additional studies are needed to verify whether the patterns we describe here are general or an artefact of our limited sample size.

Our meta-analysis indicates that, even after decades of research on the subject, few published accounts include the necessary data needed for the quantitative synthesis of community-level responses of forest understorey plants to white-tailed deer removal. In fact, half of the data sets of community diversity, cover and abundance that we constructed for this meta-analysis did not meet our previously defined minimum requirements for reasonably robust meta-regression analyses. In addition, half of the data sets exhibited some level of publication bias, which could potentially weaken the inferences presented here. Given our understanding of the available literature, we suspect that this situation would be no better (and likely worse) for similar syntheses at other levels of biological organization. For instance, 24 of the 59 articles that made it to the final stage of our iterative selection process were ultimately excluded from this meta-analysis because they focussed on species-level rather than community-level responses to deer removal. Initially, these 24 articles may seem like a reasonable sample size for a robust species-level meta-analysis. However, few of these articles focussed on the same species or taxonomic groups. Nonetheless, we are optimistic that more synthesis opportunities are available from existing data sets. Local management concerns or the evaluation of specific ecological questions typically drive the foci of experiments and reporting decisions, and these experimental or reporting decisions can unknowingly make research synthesis a difficult or impossible task. We feel that greater opportunities for the synthesis of deer impacts on plants could arise if more authors submitted their raw data as appendices to their manuscripts.

Given some critiques of exclosure experiments (Côté *et al.* 2004; Tanentzap *et al.* 2012; Frerker *et al.* 2014), it is tempting to suggest a departure from these designs and a shift towards studies employing gradients of deer density (e.g. in enclosures) to understand the magnitude and threshold levels of deer impacts. After all, a landscape completely devoid of native deer is not realistic, nor is it generally practical or desirable (Tanentzap *et al.* 2012). However, erecting numerous exclosures under similar environmental conditions is easy and economical, whereas manipulating (or finding) a gradient of deer densities in many areas is potentially more difficult and costly. Also, at some point, we must accept that hunting is an often woefully inadequate means of deer density management in many areas of North America due to a combination of sociopolitical conflicts and a heterogeneous matrix of hunter-accessible land nested within the management landscape (VerCauteren *et al.* 2011; Johnson and Horowitz 2014; Urbanek *et al.* 2015). Therefore, in some circumstances, a shifting mosaic of temporary deer exclosures may be the most appropriate management tool for maintaining the health of forest ecosystems, and we should continue to evaluate their economic feasibility and management efficacy in a variety of contexts (VerCauteren *et al*. 2006). That said, there is also an obvious need for additional well-replicated experiments that evaluate the response of plant communities against a backdrop of varying deer density (e.g. Horsley *et al*. 2003). Without these types of studies, we lack the ability to make robust management recommendations for target levels of deer density to ensure the integrity of forest ecosystems in North America.

## Conclusions

This meta-analysis is the first quantitative synthesis to indicate that the negative impacts of white-tailed deer in North American forests are significant, but not ubiquitous across all components of the forest understorey plant community. Clearly, deer exert strong negative impacts on woody plant species, and there is appropriate scientific concern that white-tailed deer overabundance is contributing to the degradation of many forested areas of North America (Côté *et al.* 2004). Community-level impacts of deer overabundance on the herbaceous understorey, however, are less clear, potentially because traditional indices of diversity may be inadequate measures of change, i.e. they do not account for potentially important compositional shifts in plant communities. To compensate for this deficiency in the literature, we suggest that previous data sets and future experiments specifically evaluate the potential for shifts in plant species identity in areas with differing levels of deer density. Also, we urge researchers to make existing and future data sets available in digital repositories to facilitate future synthesis projects related to the impacts of white-tailed deer on forest ecosystems.

## Contributions by the Authors

C.W.H. conceived, designed and conducted all statistical analysis associated with the experiment, and wrote the manuscript. A.K.S. collected the data, created the database and provided comments on the manuscript.

## Conflict of Interest Statement

None declared.

## Supporting Information

The following additional information is available in the online version of this article –

**Table S1.** Published articles included in this meta-analysis, with associated data.

**Table S2.** R code used and output produced for this meta-analysis.

Additional Information
