# Supplementary material for: Community-level impacts of white-tailed deer on understorey plants in North American forests: a meta-analysis
Source: AoB Plants. 2015 Oct 20;7:plv119. doi: 10.1093/aobpla/plv119 (PMC4676796; doi:10.1093/aobpla/plv119)
Supplement: Additional Information [file supp_7_plv119_index.html]

Community-level impacts of white-tailed deer on understory plants in North American forests: a meta-analysis — Community-level impacts of white-tailed deer on understorey plants in North American forests: a meta-analysis — Additional Information 

# Community-level impacts of white-tailed deer on understorey plants in North American forests: a meta-analysis

## Additional Information

Additional Information

- Additional Information File 1 - xlsx file
- Additional Information File 2 - doc file
