# Supplementary material for: Community-level impacts of white-tailed deer on understorey plants in North American forests: a meta-analysis
Source: AoB Plants. 2015 Oct 20;7:plv119. doi: 10.1093/aobpla/plv119 (PMC4676796; doi:10.1093/aobpla/plv119)

# Deer_meta-analysis.R

# abund.wood = import()
# cov.all = import()
# cov.herb = import()
# cov.wood = import()
# div.all = import()
# div.herb = import()
# div.wood = import()
# rich.all = import()
# rich.herb = import()
# rich.wood = import()

library(metafor)

## Loading required package: Matrix
## Loading 'metafor' package (version 1.9-6). For an overview
## and introduction to the package please type: help(metafor).

# ______________________________
abund.wood.ef = escalc(measure = "SMD", m1i = t, m2i = c, sd1i = tv, sd2i = cv, n1i = tn, n2i = cn, data = abund.wood)
test.abund.wood = rma.mv(yi,vi,random = ~1|locale, data = abund.wood.ef)
summary(test.abund.wood)

##
## Multivariate Meta-Analysis Model (k = 7; method: REML)
##
## logLik Deviance AIC BIC AICc
## -6.0502 12.1004 16.1004 15.6839 20.1004
##
## Variance Components:
##
## estim sqrt nlvls fixed factor
## sigma^2 0.0021 0.0455 6 no locale
##
## Test for Heterogeneity:
## Q(df = 6) = 6.4887, p-val = 0.3707
##
## Model Results:
##
## estimate se zval pval ci.lb ci.ub
## 0.8982 0.2152 4.1732 <.0001 0.4763 1.3200 ***
##
## ---
## Signif. codes: 0 '***' 0.001 '**' 0.01 '*' 0.05 '.' 0.1 ' ' 1

bias.abund.wood = rma.mv(yi,vi,mods = ~sqrt(vi), random = ~1|locale, data = abund.wood.ef)
summary(bias.abund.wood)

##
## Multivariate Meta-Analysis Model (k = 7; method: REML)
##
## logLik Deviance AIC BIC AICc
## -5.2847 10.5694 16.5694 15.3977 40.5694
##
## Variance Components:
##
## estim sqrt nlvls fixed factor
## sigma^2 0.0500 0.2236 6 no locale
##
## Test for Residual Heterogeneity:
## QE(df = 5) = 5.6295, p-val = 0.3440
##
## Test of Moderators (coefficient(s) 2):
## QM(df = 1) = 0.6569, p-val = 0.4177
##
## Model Results:
##
## estimate se zval pval ci.lb ci.ub
## intrcpt 1.4860 0.8125 1.8290 0.0674 -0.1064 3.0785 .
## sqrt(vi) -1.0960 1.3523 -0.8105 0.4177 -3.7465 1.5545
##
## ---
## Signif. codes: 0 '***' 0.001 '**' 0.01 '*' 0.05 '.' 0.1 ' ' 1

forest(test.abund.wood, slab = abund.wood.ef$author, annotate= FALSE, psize = 1.5, pch = 21,
 mlab = "Overall Mean Effect", addcred = TRUE, order = "obs")


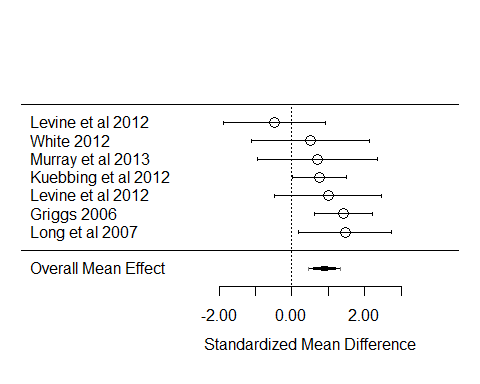


# RULE:If rstandard >3 AND hatvalue >2 times average of hatvalues,
# run analysis with those cases deleted to test for sensitivity.
rs.abund.wood = rstandard(test.abund.wood)
hat.abund.wood = hatvalues(test.abund.wood)/mean(hatvalues(test.abund.wood))
plot(hat.abund.wood, rs.abund.wood$resid, ylim = c(-4.0,4))
text(hat.abund.wood, rs.abund.wood$resid, labels = abund.wood.ef$ID, cex= 1, pos = 2)
abline(h = -3)
abline(h = 3)
abline( v = 2)


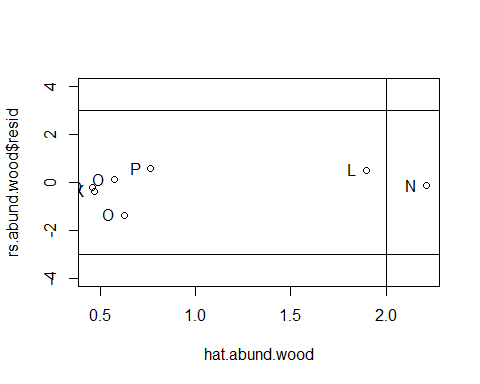


# ______________________________
cov.all.ef = escalc(measure = "SMD", m1i = t, m2i = c, sd1i = tv, sd2i = cv, n1i = tn, n2i = cn, data = cov.all)
test.cov.all = rma.mv(yi,vi,random = ~1|locale, data = cov.all.ef)
summary(test.cov.all)

##
## Multivariate Meta-Analysis Model (k = 23; method: REML)
##
## logLik Deviance AIC BIC AICc
## -93.7059 187.4118 191.4118 193.5939 192.0434
##
## Variance Components:
##
## estim sqrt nlvls fixed factor
## sigma^2 5.0675 2.2511 6 no locale
##
## Test for Heterogeneity:
## Q(df = 22) = 358.0748, p-val < .0001
##
## Model Results:
##
## estimate se zval pval ci.lb ci.ub
## 1.8284 0.9485 1.9276 0.0539 -0.0307 3.6875 .
##
## ---
## Signif. codes: 0 '***' 0.001 '**' 0.01 '*' 0.05 '.' 0.1 ' ' 1

bias.cov.all = rma.mv(yi,vi,mod = ~sqrt(vi), random = ~1|locale, data = cov.all.ef)
summary(bias.cov.all)

##
## Multivariate Meta-Analysis Model (k = 23; method: REML)
##
## logLik Deviance AIC BIC AICc
## -58.9062 117.8124 123.8124 126.9460 125.2242
##
## Variance Components:
##
## estim sqrt nlvls fixed factor
## sigma^2 17.1851 4.1455 6 no locale
##
## Test for Residual Heterogeneity:
## QE(df = 21) = 356.2657, p-val < .0001
##
## Test of Moderators (coefficient(s) 2):
## QM(df = 1) = 68.8532, p-val < .0001
##
## Model Results:
##
## estimate se zval pval ci.lb ci.ub
## intrcpt -6.5081 1.9833 -3.2815 0.0010 -10.3952 -2.6210 **
## sqrt(vi) 12.0675 1.4543 8.2978 <.0001 9.2171 14.9179 ***
##
## ---
## Signif. codes: 0 '***' 0.001 '**' 0.01 '*' 0.05 '.' 0.1 ' ' 1

forest(test.cov.all, slab = cov.all.ef$author, annotate= FALSE, psize = 1.5, pch = 21,
 mlab = "Overall Mean Effect", addcred = TRUE, order = "obs")


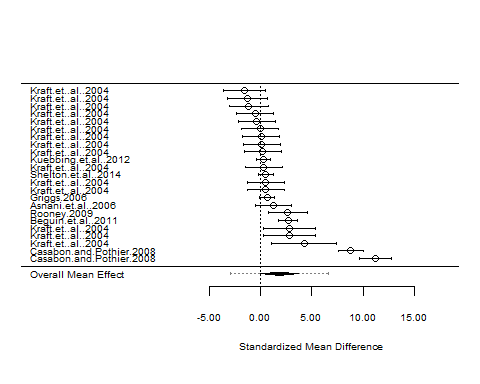


rs.cov.all = rstandard(test.cov.all)
hat.cov.all = hatvalues(test.cov.all)/mean(hatvalues(test.cov.all))
plot(hat.cov.all, rs.cov.all$resid, ylim = c(-4.0,10))
text(hat.cov.all, rs.cov.all$resid, labels = cov.all.ef$ID, cex= 1, pos = 2)
abline(h = -3)
abline(h = 3)
abline( v = 2)


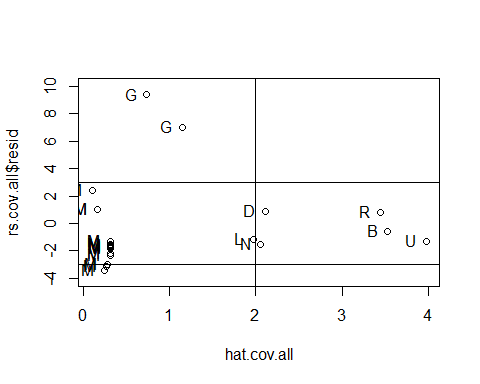


mod1.cov.all = rma.mv(yi,vi,mods = deer.km2, random = ~1|locale, data = cov.all.ef)

## Warning in rma.mv(yi, vi, mods = deer.km2, random = ~1 | locale, data =
## cov.all.ef): Rows with NAs omitted from model fitting.

summary(mod1.cov.all)

##
## Multivariate Meta-Analysis Model (k = 21; method: REML)
##
## logLik Deviance AIC BIC AICc
## -43.4175 86.8350 92.8350 95.6683 94.4350
##
## Variance Components:
##
## estim sqrt nlvls fixed factor
## sigma^2 185.8731 13.6335 5 no locale
##
## Test for Residual Heterogeneity:
## QE(df = 19) = 332.5584, p-val < .0001
##
## Test of Moderators (coefficient(s) 2):
## QM(df = 1) = 101.0100, p-val < .0001
##
## Model Results:
##
## estimate se zval pval ci.lb ci.ub
## intrcpt 30.9786 6.7445 4.5932 <.0001 17.7597 44.1976 ***
## mods -1.3582 0.1351 -10.0504 <.0001 -1.6231 -1.0933 ***
##
## ---
## Signif. codes: 0 '***' 0.001 '**' 0.01 '*' 0.05 '.' 0.1 ' ' 1

mod2.cov.all = rma.mv(yi,vi,mods = plot.m2, random = ~1|locale, data = cov.all.ef)
summary(mod2.cov.all)

##
## Multivariate Meta-Analysis Model (k = 23; method: REML)
##
## logLik Deviance AIC BIC AICc
## -91.5759 183.1519 189.1519 192.2854 190.5636
##
## Variance Components:
##
## estim sqrt nlvls fixed factor
## sigma^2 7.7231 2.7791 6 no locale
##
## Test for Residual Heterogeneity:
## QE(df = 21) = 357.6168, p-val < .0001
##
## Test of Moderators (coefficient(s) 2):
## QM(df = 1) = 0.4881, p-val = 0.4848
##
## Model Results:
##
## estimate se zval pval ci.lb ci.ub
## intrcpt 1.3649 1.3383 1.0199 0.3078 -1.2581 3.9879
## mods 0.0006 0.0008 0.6986 0.4848 -0.0010 0.0022
##
## ---
## Signif. codes: 0 '***' 0.001 '**' 0.01 '*' 0.05 '.' 0.1 ' ' 1

mod3.cov.all = rma.mv(yi,vi,mods = yrs, random = ~1|locale, data = cov.all.ef)
summary(mod3.cov.all)

##
## Multivariate Meta-Analysis Model (k = 23; method: REML)
##
## logLik Deviance AIC BIC AICc
## -92.3002 184.6004 190.6004 193.7340 192.0122
##
## Variance Components:
##
## estim sqrt nlvls fixed factor
## sigma^2 4.9615 2.2274 6 no locale
##
## Test for Residual Heterogeneity:
## QE(df = 21) = 346.6583, p-val < .0001
##
## Test of Moderators (coefficient(s) 2):
## QM(df = 1) = 0.6024, p-val = 0.4377
##
## Model Results:
##
## estimate se zval pval ci.lb ci.ub
## intrcpt 1.2933 1.1648 1.1103 0.2669 -0.9897 3.5764
## mods 0.0622 0.0801 0.7761 0.4377 -0.0949 0.2192
##
## ---
## Signif. codes: 0 '***' 0.001 '**' 0.01 '*' 0.05 '.' 0.1 ' ' 1

# ______________________________
cov.wood.ef = escalc(measure = "SMD", m1i = t, m2i = c, sd1i = tv, sd2i = cv, n1i = tn, n2i = cn, data = cov.wood)
test.cov.wood = rma.mv(yi,vi,random = ~1|locale, data = cov.wood.ef)
summary(test.cov.wood)

##
## Multivariate Meta-Analysis Model (k = 8; method: REML)
##
## logLik Deviance AIC BIC AICc
## -5.2306 10.4612 14.4612 14.3530 17.4612
##
## Variance Components:
##
## estim sqrt nlvls fixed factor
## sigma^2 0.1956 0.4422 5 no locale
##
## Test for Heterogeneity:
## Q(df = 7) = 12.1761, p-val = 0.0949
##
## Model Results:
##
## estimate se zval pval ci.lb ci.ub
## 1.2428 0.2497 4.9774 <.0001 0.7534 1.7321 ***
##
## ---
## Signif. codes: 0 '***' 0.001 '**' 0.01 '*' 0.05 '.' 0.1 ' ' 1

bias.cov.wood = rma.mv(yi,vi,mod = ~sqrt(vi), random = ~1|locale, data = cov.wood.ef)
summary(bias.cov.wood)

##
## Multivariate Meta-Analysis Model (k = 8; method: REML)
##
## logLik Deviance AIC BIC AICc
## -4.5778 9.1557 15.1557 14.5310 27.1557
##
## Variance Components:
##
## estim sqrt nlvls fixed factor
## sigma^2 0.2966 0.5446 5 no locale
##
## Test for Residual Heterogeneity:
## QE(df = 6) = 10.4929, p-val = 0.1054
##
## Test of Moderators (coefficient(s) 2):
## QM(df = 1) = 0.0471, p-val = 0.8281
##
## Model Results:
##
## estimate se zval pval ci.lb ci.ub
## intrcpt 1.5278 1.2825 1.1913 0.2336 -0.9858 4.0414
## sqrt(vi) -0.6881 3.1693 -0.2171 0.8281 -6.8998 5.5236
##
## ---
## Signif. codes: 0 '***' 0.001 '**' 0.01 '*' 0.05 '.' 0.1 ' ' 1

forest(test.cov.wood,slab = cov.wood.ef$author, annotate= FALSE, psize = 1.5, pch = 21,
 mlab = "Overall Mean Effect", addcred = TRUE, order = "obs")


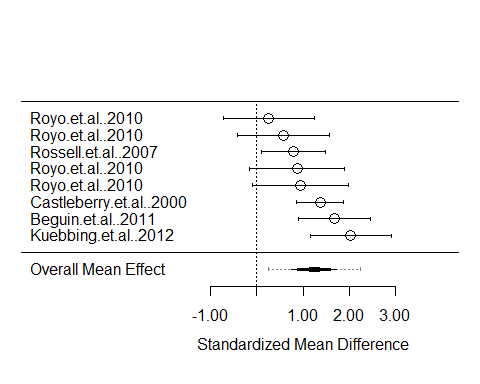


rs.cov.wood = rstandard(test.cov.wood)
hat.cov.wood = hatvalues(test.cov.wood)/mean(hatvalues(test.cov.wood))
plot(hat.cov.wood, rs.cov.wood$resid, ylim = c(-4,4))
text(hat.cov.wood, rs.cov.wood$resid, labels = cov.wood.ef$ID, cex= 1, pos = 2)
abline(h = -3)
abline(h = 3)
abline(v = 2)


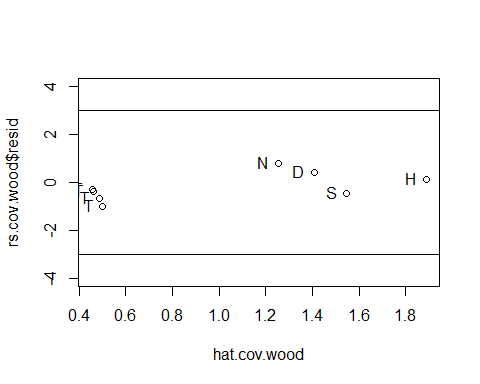


# ______________________________
cov.herb.ef = escalc(measure = "SMD", m1i = t, m2i = c, sd1i = tv, sd2i = cv, n1i = tn, n2i = cn, data = cov.herb)
test.cov.herb = rma.mv(yi,vi,random = ~1|locale, data = cov.herb.ef)
summary(test.cov.herb)

##
## Multivariate Meta-Analysis Model (k = 14; method: REML)
##
## logLik Deviance AIC BIC AICc
## -15.8640 31.7280 35.7280 36.8579 36.9280
##
## Variance Components:
##
## estim sqrt nlvls fixed factor
## sigma^2 1.2533 1.1195 8 no locale
##
## Test for Heterogeneity:
## Q(df = 13) = 97.8833, p-val < .0001
##
## Model Results:
##
## estimate se zval pval ci.lb ci.ub
## 0.6609 0.4229 1.5629 0.1181 -0.1679 1.4898
##
## ---
## Signif. codes: 0 '***' 0.001 '**' 0.01 '*' 0.05 '.' 0.1 ' ' 1

bias.cov.herb = rma.mv(yi,vi,mod = ~sqrt(vi), random = ~1|locale, data = cov.herb.ef)
summary(bias.cov.herb)

##
## Multivariate Meta-Analysis Model (k = 14; method: REML)
##
## logLik Deviance AIC BIC AICc
## -12.7712 25.5424 31.5424 32.9971 34.5424
##
## Variance Components:
##
## estim sqrt nlvls fixed factor
## sigma^2 0.9416 0.9704 8 no locale
##
## Test for Residual Heterogeneity:
## QE(df = 12) = 67.5777, p-val < .0001
##
## Test of Moderators (coefficient(s) 2):
## QM(df = 1) = 4.0322, p-val = 0.0446
##
## Model Results:
##
## estimate se zval pval ci.lb ci.ub
## intrcpt -1.1786 0.9813 -1.2011 0.2297 -3.1020 0.7447
## sqrt(vi) 4.1122 2.0479 2.0080 0.0446 0.0984 8.1260 *
##
## ---
## Signif. codes: 0 '***' 0.001 '**' 0.01 '*' 0.05 '.' 0.1 ' ' 1

forest(test.cov.herb,slab = cov.herb.ef$author, annotate= FALSE, psize = 1.5, pch = 21,
 mlab = "Overall Mean Effect", addcred = TRUE, order = "obs")


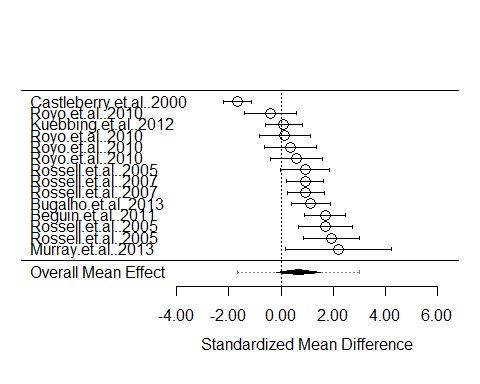


rs.cov.herb = rstandard(test.cov.herb)
hat.cov.herb = hatvalues(test.cov.herb)/mean(hatvalues(test.cov.herb))
plot(hat.cov.herb, rs.cov.herb$resid, ylim = c(-10,10))
text(hat.cov.herb, rs.cov.herb$resid, labels = cov.herb.ef$ID, cex= 1, pos = 2)
abline(h = -3)
abline(h = 3)
abline(v = 2)


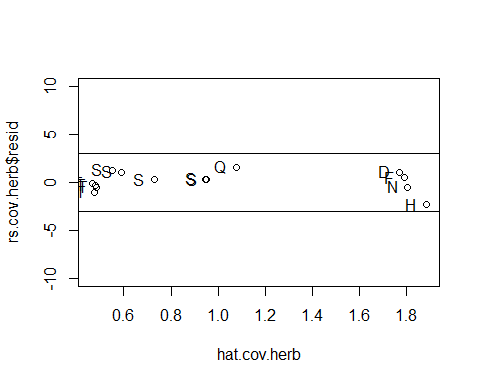


mod1.cov.herb = rma.mv(yi,vi,mods = deer.km2, random = ~1|locale, data = cov.herb.ef)

## Warning in rma.mv(yi, vi, mods = deer.km2, random = ~1 | locale, data =
## cov.herb.ef): Rows with NAs omitted from model fitting.

summary(mod1.cov.herb)

##
## Multivariate Meta-Analysis Model (k = 12; method: REML)
##
## logLik Deviance AIC BIC AICc
## -10.4562 20.9124 26.9124 27.8202 30.9124
##
## Variance Components:
##
## estim sqrt nlvls fixed factor
## sigma^2 1.3223 1.1499 6 no locale
##
## Test for Residual Heterogeneity:
## QE(df = 10) = 63.7505, p-val < .0001
##
## Test of Moderators (coefficient(s) 2):
## QM(df = 1) = 1.4463, p-val = 0.2291
##
## Model Results:
##
## estimate se zval pval ci.lb ci.ub
## intrcpt 0.0239 0.6841 0.0349 0.9721 -1.3170 1.3648
## mods 0.0270 0.0225 1.2026 0.2291 -0.0170 0.0711
##
## ---
## Signif. codes: 0 '***' 0.001 '**' 0.01 '*' 0.05 '.' 0.1 ' ' 1

mod2.cov.herb = rma.mv(yi,vi,mods = plot.m2, random = ~1|locale, data = cov.herb.ef)
summary(mod2.cov.herb)

##
## Multivariate Meta-Analysis Model (k = 14; method: REML)
##
## logLik Deviance AIC BIC AICc
## -14.1619 28.3238 34.3238 35.7785 37.3238
##
## Variance Components:
##
## estim sqrt nlvls fixed factor
## sigma^2 1.4648 1.2103 8 no locale
##
## Test for Residual Heterogeneity:
## QE(df = 12) = 95.8670, p-val < .0001
##
## Test of Moderators (coefficient(s) 2):
## QM(df = 1) = 0.0954, p-val = 0.7574
##
## Model Results:
##
## estimate se zval pval ci.lb ci.ub
## intrcpt 0.7694 0.5572 1.3809 0.1673 -0.3226 1.8615
## mods -0.0010 0.0034 -0.3089 0.7574 -0.0077 0.0056
##
## ---
## Signif. codes: 0 '***' 0.001 '**' 0.01 '*' 0.05 '.' 0.1 ' ' 1

mod3.cov.herb = rma.mv(yi,vi,mods = yrs, random = ~1|locale, data = cov.herb.ef)
summary(mod3.cov.herb)

##
## Multivariate Meta-Analysis Model (k = 14; method: REML)
##
## logLik Deviance AIC BIC AICc
## -14.7405 29.4811 35.4811 36.9358 38.4811
##
## Variance Components:
##
## estim sqrt nlvls fixed factor
## sigma^2 1.4744 1.2142 8 no locale
##
## Test for Residual Heterogeneity:
## QE(df = 12) = 96.4738, p-val < .0001
##
## Test of Moderators (coefficient(s) 2):
## QM(df = 1) = 0.0005, p-val = 0.9828
##
## Model Results:
##
## estimate se zval pval ci.lb ci.ub
## intrcpt 0.6530 0.9038 0.7226 0.4699 -1.1183 2.4244
## mods 0.0030 0.1376 0.0215 0.9828 -0.2667 0.2726
##
## ---
## Signif. codes: 0 '***' 0.001 '**' 0.01 '*' 0.05 '.' 0.1 ' ' 1

# ______________________________
rich.all.ef = escalc(measure = "SMD", m1i = t, m2i = c, sd1i = tv, sd2i = cv, n1i = tn, n2i = cn, data = rich.all)
test.rich.all = rma.mv(yi,vi,random = ~1|locale, data = rich.all.ef)
summary(test.rich.all)

##
## Multivariate Meta-Analysis Model (k = 14; method: REML)
##
## logLik Deviance AIC BIC AICc
## -42.6679 85.3359 89.3359 90.4658 90.5359
##
## Variance Components:
##
## estim sqrt nlvls fixed factor
## sigma^2 0.6790 0.8240 6 no locale
##
## Test for Heterogeneity:
## Q(df = 13) = 102.3478, p-val < .0001
##
## Model Results:
##
## estimate se zval pval ci.lb ci.ub
## 0.3381 0.3749 0.9020 0.3670 -0.3966 1.0729
##
## ---
## Signif. codes: 0 '***' 0.001 '**' 0.01 '*' 0.05 '.' 0.1 ' ' 1

bias.rich.all = rma.mv(yi,vi,mod = ~sqrt(vi), random = ~1|locale, data = rich.all.ef)
summary(bias.rich.all)

##
## Multivariate Meta-Analysis Model (k = 14; method: REML)
##
## logLik Deviance AIC BIC AICc
## -37.1725 74.3449 80.3449 81.7997 83.3449
##
## Variance Components:
##
## estim sqrt nlvls fixed factor
## sigma^2 1.4747 1.2144 6 no locale
##
## Test for Residual Heterogeneity:
## QE(df = 12) = 101.6992, p-val < .0001
##
## Test of Moderators (coefficient(s) 2):
## QM(df = 1) = 11.0366, p-val = 0.0009
##
## Model Results:
##
## estimate se zval pval ci.lb ci.ub
## intrcpt 1.9924 0.7215 2.7616 0.0058 0.5783 3.4065 **
## sqrt(vi) -3.0532 0.9190 -3.3221 0.0009 -4.8545 -1.2519 ***
##
## ---
## Signif. codes: 0 '***' 0.001 '**' 0.01 '*' 0.05 '.' 0.1 ' ' 1

forest(test.rich.all,slab = rich.all.ef$author, annotate= FALSE, psize = 1.5, pch = 21,
 mlab = "Overall Mean Effect", addcred = TRUE, order = "obs")


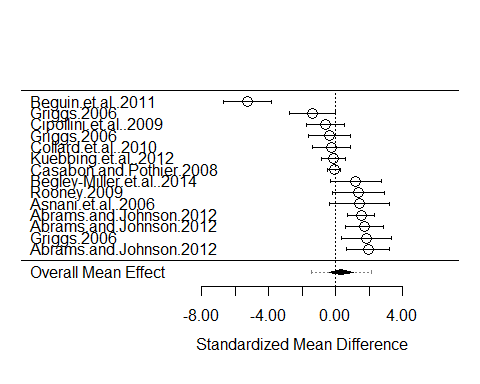


rs.rich.all = rstandard(test.rich.all)
hat.rich.all = hatvalues(test.rich.all)/mean(hatvalues(test.rich.all))
plot(hat.rich.all, rs.rich.all$resid, ylim = c(-10,10))
text(hat.rich.all, rs.rich.all$resid, labels = rich.all.ef$ID, cex= 1, pos = 2)
abline(h = -3)
abline(h = 3)
abline(v = 2)


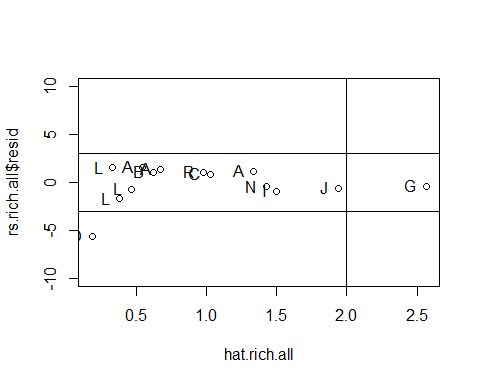


mod1.rich.all = rma.mv(yi,vi,mods = deer.km2, random = ~1|locale, data = rich.all.ef)

## Warning in rma.mv(yi, vi, mods = deer.km2, random = ~1 | locale, data =
## rich.all.ef): Rows with NAs omitted from model fitting.

summary(mod1.rich.all)

##
## Multivariate Meta-Analysis Model (k = 13; method: REML)
##
## logLik Deviance AIC BIC AICc
## -39.9380 79.8760 85.8760 87.0697 89.3046
##
## Variance Components:
##
## estim sqrt nlvls fixed factor
## sigma^2 0.0257 0.1604 6 no locale
##
## Test for Residual Heterogeneity:
## QE(df = 11) = 70.0268, p-val < .0001
##
## Test of Moderators (coefficient(s) 2):
## QM(df = 1) = 21.7993, p-val < .0001
##
## Model Results:
##
## estimate se zval pval ci.lb ci.ub
## intrcpt -0.6442 0.2478 -2.5994 0.0093 -1.1300 -0.1585 **
## mods 0.0248 0.0053 4.6690 <.0001 0.0144 0.0352 ***
##
## ---
## Signif. codes: 0 '***' 0.001 '**' 0.01 '*' 0.05 '.' 0.1 ' ' 1

mod2.rich.all = rma.mv(yi,vi,mods = plot.m2, random = ~1|locale, data = rich.all.ef)
summary(mod2.rich.all)

##
## Multivariate Meta-Analysis Model (k = 14; method: REML)
##
## logLik Deviance AIC BIC AICc
## -40.0347 80.0695 86.0695 87.5242 89.0695
##
## Variance Components:
##
## estim sqrt nlvls fixed factor
## sigma^2 0.8093 0.8996 6 no locale
##
## Test for Residual Heterogeneity:
## QE(df = 12) = 100.1568, p-val < .0001
##
## Test of Moderators (coefficient(s) 2):
## QM(df = 1) = 3.4927, p-val = 0.0616
##
## Model Results:
##
## estimate se zval pval ci.lb ci.ub
## intrcpt 0.1459 0.4164 0.3504 0.7260 -0.6702 0.9620
## mods 0.0005 0.0003 1.8689 0.0616 -0.0000 0.0010 .
##
## ---
## Signif. codes: 0 '***' 0.001 '**' 0.01 '*' 0.05 '.' 0.1 ' ' 1

mod3.rich.all = rma.mv(yi,vi,mods = yrs, random = ~1|locale, data = rich.all.ef)
summary(mod3.rich.all)

##
## Multivariate Meta-Analysis Model (k = 14; method: REML)
##
## logLik Deviance AIC BIC AICc
## -36.8874 73.7747 79.7747 81.2295 82.7747
##
## Variance Components:
##
## estim sqrt nlvls fixed factor
## sigma^2 0.0719 0.2681 6 no locale
##
## Test for Residual Heterogeneity:
## QE(df = 12) = 64.6821, p-val < .0001
##
## Test of Moderators (coefficient(s) 2):
## QM(df = 1) = 20.4700, p-val < .0001
##
## Model Results:
##
## estimate se zval pval ci.lb ci.ub
## intrcpt -1.4582 0.4210 -3.4635 0.0005 -2.2834 -0.6330 ***
## mods 0.1580 0.0349 4.5244 <.0001 0.0896 0.2265 ***
##
## ---
## Signif. codes: 0 '***' 0.001 '**' 0.01 '*' 0.05 '.' 0.1 ' ' 1

# ______________________________
rich.wood.ef = escalc(measure = "SMD", m1i = t, m2i = c, sd1i = tv, sd2i = cv, n1i = tn, n2i = cn, data = rich.wood)
test.rich.wood = rma.mv(yi,vi,random = ~1|locale, data = rich.wood.ef)
summary(test.rich.wood)

##
## Multivariate Meta-Analysis Model (k = 19; method: REML)
##
## logLik Deviance AIC BIC AICc
## -45.3192 90.6384 94.6384 96.4191 95.4384
##
## Variance Components:
##
## estim sqrt nlvls fixed factor
## sigma^2 0.4776 0.6911 11 no locale
##
## Test for Heterogeneity:
## Q(df = 18) = 93.4815, p-val < .0001
##
## Model Results:
##
## estimate se zval pval ci.lb ci.ub
## 0.6979 0.2515 2.7755 0.0055 0.2051 1.1908 **
##
## ---
## Signif. codes: 0 '***' 0.001 '**' 0.01 '*' 0.05 '.' 0.1 ' ' 1

bias.rich.wood = rma.mv(yi,vi,mod = ~sqrt(vi), random = ~1|locale, data = rich.wood.ef)
summary(bias.rich.wood)

##
## Multivariate Meta-Analysis Model (k = 19; method: REML)
##
## logLik Deviance AIC BIC AICc
## -21.5710 43.1419 49.1419 51.6416 50.9881
##
## Variance Components:
##
## estim sqrt nlvls fixed factor
## sigma^2 0.5443 0.7377 11 no locale
##
## Test for Residual Heterogeneity:
## QE(df = 17) = 41.1190, p-val = 0.0009
##
## Test of Moderators (coefficient(s) 2):
## QM(df = 1) = 43.6625, p-val < .0001
##
## Model Results:
##
## estimate se zval pval ci.lb ci.ub
## intrcpt -1.3119 0.4031 -3.2544 0.0011 -2.1019 -0.5218 **
## sqrt(vi) 4.0800 0.6175 6.6078 <.0001 2.8698 5.2902 ***
##
## ---
## Signif. codes: 0 '***' 0.001 '**' 0.01 '*' 0.05 '.' 0.1 ' ' 1

forest(test.rich.wood,slab = rich.wood.ef$author, annotate= FALSE, psize = 1.5, pch = 21,
 mlab = "Overall Mean Effect", addcred = TRUE, order = "obs")


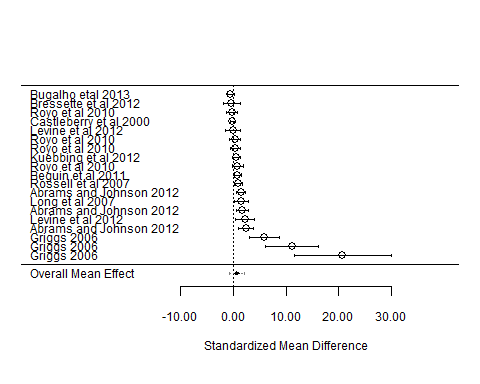


rs.rich.wood = rstandard(test.rich.wood)
hat.rich.wood = hatvalues(test.rich.wood)/mean(hatvalues(test.rich.wood))
plot(hat.rich.wood, rs.rich.wood$resid, ylim = c(-15,15))
text(hat.rich.wood, rs.rich.wood$resid, labels = rich.wood.ef$ID, cex= 1, pos = 2)
abline(h = -3)
abline(h = 3)
abline(v = 2)


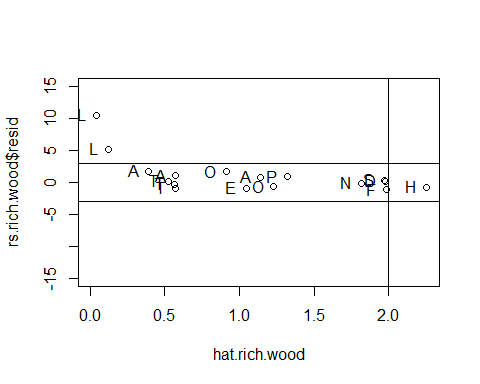


mod1.rich.wood = rma.mv(yi,vi,mods = deer.km2, random = ~1|locale, data = rich.wood.ef)

## Warning in rma.mv(yi, vi, mods = deer.km2, random = ~1 | locale, data =
## rich.wood.ef): Rows with NAs omitted from model fitting.

summary(mod1.rich.wood)

##
## Multivariate Meta-Analysis Model (k = 18; method: REML)
##
## logLik Deviance AIC BIC AICc
## -37.6370 75.2739 81.2739 83.5917 83.2739
##
## Variance Components:
##
## estim sqrt nlvls fixed factor
## sigma^2 0.2130 0.4615 10 no locale
##
## Test for Residual Heterogeneity:
## QE(df = 16) = 62.5671, p-val < .0001
##
## Test of Moderators (coefficient(s) 2):
## QM(df = 1) = 10.7754, p-val = 0.0010
##
## Model Results:
##
## estimate se zval pval ci.lb ci.ub
## intrcpt 0.0411 0.2623 0.1567 0.8755 -0.4731 0.5553
## mods 0.0267 0.0081 3.2826 0.0010 0.0107 0.0426 **
##
## ---
## Signif. codes: 0 '***' 0.001 '**' 0.01 '*' 0.05 '.' 0.1 ' ' 1

mod2.rich.wood = rma.mv(yi,vi,mods = plot.m2, random = ~1|locale, data = rich.wood.ef)
summary(mod2.rich.wood)

##
## Multivariate Meta-Analysis Model (k = 19; method: REML)
##
## logLik Deviance AIC BIC AICc
## -43.8900 87.7799 93.7799 96.2795 95.6261
##
## Variance Components:
##
## estim sqrt nlvls fixed factor
## sigma^2 0.4910 0.7007 11 no locale
##
## Test for Residual Heterogeneity:
## QE(df = 17) = 92.3117, p-val < .0001
##
## Test of Moderators (coefficient(s) 2):
## QM(df = 1) = 0.8695, p-val = 0.3511
##
## Model Results:
##
## estimate se zval pval ci.lb ci.ub
## intrcpt 0.7589 0.2621 2.8950 0.0038 0.2451 1.2726 **
## mods -0.0000 0.0000 -0.9325 0.3511 -0.0001 0.0000
##
## ---
## Signif. codes: 0 '***' 0.001 '**' 0.01 '*' 0.05 '.' 0.1 ' ' 1

mod3.rich.wood = rma.mv(yi,vi,mods = yrs, random = ~1|locale, data = rich.wood.ef)
summary(mod3.rich.wood)

##
## Multivariate Meta-Analysis Model (k = 19; method: REML)
##
## logLik Deviance AIC BIC AICc
## -45.0798 90.1597 96.1597 98.6593 98.0058
##
## Variance Components:
##
## estim sqrt nlvls fixed factor
## sigma^2 18.0909 4.2533 11 no locale
##
## Test for Residual Heterogeneity:
## QE(df = 17) = 74.5272, p-val < .0001
##
## Test of Moderators (coefficient(s) 2):
## QM(df = 1) = 18.4833, p-val < .0001
##
## Model Results:
##
## estimate se zval pval ci.lb ci.ub
## intrcpt 7.3651 2.0109 3.6625 0.0002 3.4238 11.3065 ***
## mods -0.6966 0.1620 -4.2992 <.0001 -1.0142 -0.3790 ***
##
## ---
## Signif. codes: 0 '***' 0.001 '**' 0.01 '*' 0.05 '.' 0.1 ' ' 1

# ______________________________
rich.herb.ef = escalc(measure = "SMD", m1i = t, m2i = c, sd1i = tv, sd2i = cv, n1i = tn, n2i = cn, data = rich.herb)
test.rich.herb = rma.mv(yi,vi,random = ~1|locale, data = rich.herb.ef)
summary(test.rich.herb)

##
## Multivariate Meta-Analysis Model (k = 15; method: REML)
##
## logLik Deviance AIC BIC AICc
## -19.2162 38.4325 42.4325 43.7106 43.5234
##
## Variance Components:
##
## estim sqrt nlvls fixed factor
## sigma^2 1.2951 1.1380 7 no locale
##
## Test for Heterogeneity:
## Q(df = 14) = 91.1000, p-val < .0001
##
## Model Results:
##
## estimate se zval pval ci.lb ci.ub
## 0.0521 0.4558 0.1142 0.9090 -0.8412 0.9453
##
## ---
## Signif. codes: 0 '***' 0.001 '**' 0.01 '*' 0.05 '.' 0.1 ' ' 1

bias.rich.herb = rma.mv(yi,vi,mod = ~sqrt(vi), random = ~1|locale, data = rich.herb.ef)
summary(bias.rich.herb)

##
## Multivariate Meta-Analysis Model (k = 15; method: REML)
##
## logLik Deviance AIC BIC AICc
## -16.9062 33.8123 39.8123 41.5072 42.4790
##
## Variance Components:
##
## estim sqrt nlvls fixed factor
## sigma^2 1.1321 1.0640 7 no locale
##
## Test for Residual Heterogeneity:
## QE(df = 13) = 77.9452, p-val < .0001
##
## Test of Moderators (coefficient(s) 2):
## QM(df = 1) = 3.2680, p-val = 0.0706
##
## Model Results:
##
## estimate se zval pval ci.lb ci.ub
## intrcpt -1.0173 0.7287 -1.3959 0.1627 -2.4456 0.4110
## sqrt(vi) 2.3114 1.2786 1.8078 0.0706 -0.1946 4.8174 .
##
## ---
## Signif. codes: 0 '***' 0.001 '**' 0.01 '*' 0.05 '.' 0.1 ' ' 1

forest(test.rich.herb,slab = rich.herb.ef$author, annotate= FALSE, psize = 1.5, pch = 21,
 mlab = "Overall Mean Effect", addcred = TRUE, order = "obs")


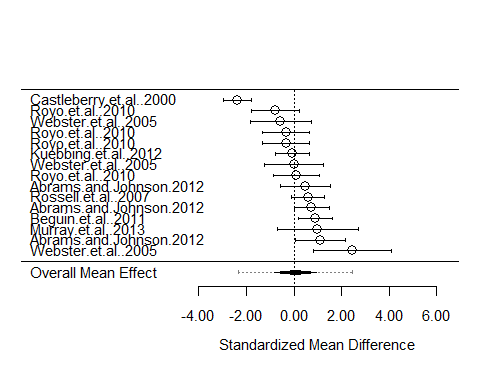


rs.rich.herb = rstandard(test.rich.herb)
hat.rich.herb = hatvalues(test.rich.herb)/mean(hatvalues(test.rich.herb))
plot(hat.rich.herb, rs.rich.herb$resid, ylim = c(-5,5))
text(hat.rich.herb, rs.rich.herb$resid, labels = rich.herb.ef$ID, cex= 1, pos = 2)
abline(h = -3)
abline(h = 3)
abline(v = 2)


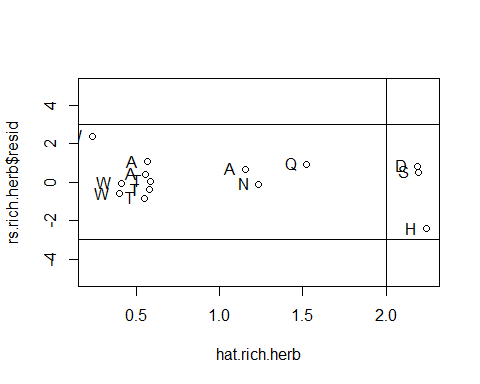


mod1.rich.herb = rma.mv(yi,vi,mods = deer.km2, random = ~1|locale, data = rich.herb.ef)

## Warning in rma.mv(yi, vi, mods = deer.km2, random = ~1 | locale, data =
## rich.herb.ef): Rows with NAs omitted from model fitting.

summary(mod1.rich.herb)

##
## Multivariate Meta-Analysis Model (k = 13; method: REML)
##
## logLik Deviance AIC BIC AICc
## -14.7856 29.5712 35.5712 36.7649 38.9998
##
## Variance Components:
##
## estim sqrt nlvls fixed factor
## sigma^2 1.2354 1.1115 6 no locale
##
## Test for Residual Heterogeneity:
## QE(df = 11) = 62.2418, p-val < .0001
##
## Test of Moderators (coefficient(s) 2):
## QM(df = 1) = 1.7344, p-val = 0.1879
##
## Model Results:
##
## estimate se zval pval ci.lb ci.ub
## intrcpt -0.7069 0.7028 -1.0058 0.3145 -2.0844 0.6706
## mods 0.0238 0.0180 1.3170 0.1879 -0.0116 0.0591
##
## ---
## Signif. codes: 0 '***' 0.001 '**' 0.01 '*' 0.05 '.' 0.1 ' ' 1

mod2.rich.herb = rma.mv(yi,vi,mods = plot.m2, random = ~1|locale, data = rich.herb.ef)
summary(mod2.rich.herb)

##
## Multivariate Meta-Analysis Model (k = 15; method: REML)
##
## logLik Deviance AIC BIC AICc
## -17.5532 35.1064 41.1064 42.8013 43.7731
##
## Variance Components:
##
## estim sqrt nlvls fixed factor
## sigma^2 1.5777 1.2561 7 no locale
##
## Test for Residual Heterogeneity:
## QE(df = 13) = 89.8982, p-val < .0001
##
## Test of Moderators (coefficient(s) 2):
## QM(df = 1) = 0.0225, p-val = 0.8807
##
## Model Results:
##
## estimate se zval pval ci.lb ci.ub
## intrcpt 0.1156 0.6286 0.1840 0.8540 -1.1163 1.3476
## mods -0.0005 0.0036 -0.1501 0.8807 -0.0075 0.0065
##
## ---
## Signif. codes: 0 '***' 0.001 '**' 0.01 '*' 0.05 '.' 0.1 ' ' 1

mod3.rich.herb = rma.mv(yi,vi,mods = yrs, random = ~1|locale, data = rich.herb.ef)
summary(mod3.rich.herb)

##
## Multivariate Meta-Analysis Model (k = 15; method: REML)
##
## logLik Deviance AIC BIC AICc
## -17.9370 35.8740 41.8740 43.5688 44.5406
##
## Variance Components:
##
## estim sqrt nlvls fixed factor
## sigma^2 1.3421 1.1585 7 no locale
##
## Test for Residual Heterogeneity:
## QE(df = 13) = 68.0281, p-val < .0001
##
## Test of Moderators (coefficient(s) 2):
## QM(df = 1) = 0.0688, p-val = 0.7931
##
## Model Results:
##
## estimate se zval pval ci.lb ci.ub
## intrcpt -0.0914 0.7200 -0.1269 0.8990 -1.5027 1.3199
## mods 0.0185 0.0706 0.2623 0.7931 -0.1199 0.1569
##
## ---
## Signif. codes: 0 '***' 0.001 '**' 0.01 '*' 0.05 '.' 0.1 ' ' 1

# ______________________________
div.all.ef = escalc(measure = "SMD", m1i = t, m2i = c, sd1i = tv, sd2i = cv, n1i = tn, n2i = cn, data = div.all)
test.div.all = rma.mv(yi,vi,random = ~1|locale, data = div.all.ef)
summary(test.div.all)

##
## Multivariate Meta-Analysis Model (k = 7; method: REML)
##
## logLik Deviance AIC BIC AICc
## -16.2190 32.4380 36.4380 36.0215 40.4380
##
## Variance Components:
##
## estim sqrt nlvls fixed factor
## sigma^2 0.8452 0.9193 3 no locale
##
## Test for Heterogeneity:
## Q(df = 6) = 25.8401, p-val = 0.0002
##
## Model Results:
##
## estimate se zval pval ci.lb ci.ub
## 0.7730 0.6265 1.2340 0.2172 -0.4548 2.0009
##
## ---
## Signif. codes: 0 '***' 0.001 '**' 0.01 '*' 0.05 '.' 0.1 ' ' 1

bias.div.all = rma.mv(yi,vi,mod = ~sqrt(vi), random = ~1|locale, data = div.all.ef)
summary(bias.div.all)

##
## Multivariate Meta-Analysis Model (k = 7; method: REML)
##
## logLik Deviance AIC BIC AICc
## -15.8213 31.6425 37.6425 36.4709 61.6425
##
## Variance Components:
##
## estim sqrt nlvls fixed factor
## sigma^2 0.9591 0.9793 3 no locale
##
## Test for Residual Heterogeneity:
## QE(df = 5) = 25.8304, p-val < .0001
##
## Test of Moderators (coefficient(s) 2):
## QM(df = 1) = 0.0315, p-val = 0.8591
##
## Model Results:
##
## estimate se zval pval ci.lb ci.ub
## intrcpt 0.5827 1.2077 0.4825 0.6294 -1.7842 2.9497
## sqrt(vi) 0.2483 1.3984 0.1776 0.8591 -2.4926 2.9892
##
## ---
## Signif. codes: 0 '***' 0.001 '**' 0.01 '*' 0.05 '.' 0.1 ' ' 1

forest(test.div.all,slab = div.all.ef$author, annotate= FALSE, psize = 1.5, pch = 21,
 mlab = "Overall Mean Effect", addcred = TRUE, order = "obs")


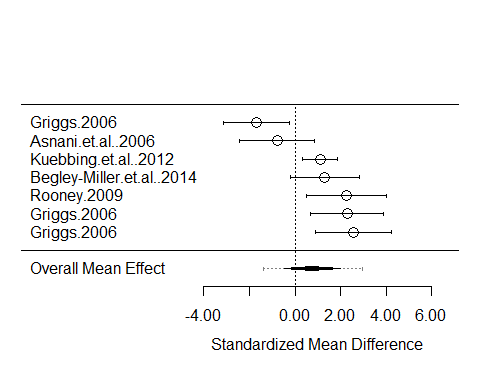


rs.div.all = rstandard(test.div.all)
hat.div.all = hatvalues(test.div.all)/mean(hatvalues(test.div.all))
plot(hat.div.all, rs.div.all$resid, ylim = c(-5,5))
text(hat.div.all, rs.div.all$resid, labels = div.all.ef$ID, cex= 1, pos = 2)
abline(h = -3)
abline(h = 3)
abline(v = 2)


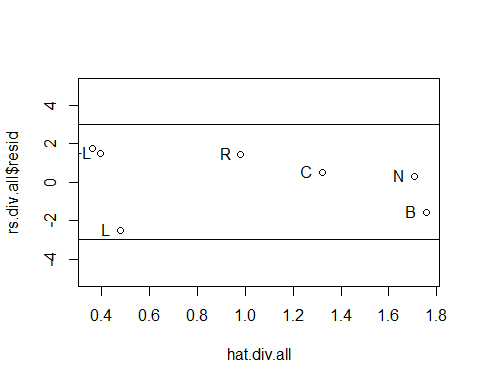


# ______________________________
div.wood.ef = escalc(measure = "SMD", m1i = t, m2i = c, sd1i = tv, sd2i = cv, n1i = tn, n2i = cn, data = div.wood)
test.div.wood = rma.mv(yi,vi,random = ~1|locale, data = div.wood.ef)
summary(test.div.wood)

##
## Multivariate Meta-Analysis Model (k = 5; method: REML)
##
## logLik Deviance AIC BIC AICc
## -8.3344 16.6689 20.6689 19.4415 32.6689
##
## Variance Components:
##
## estim sqrt nlvls fixed factor
## sigma^2 10.1663 3.1885 3 no locale
##
## Test for Heterogeneity:
## Q(df = 4) = 51.8030, p-val < .0001
##
## Model Results:
##
## estimate se zval pval ci.lb ci.ub
## 1.3375 1.8637 0.7176 0.4730 -2.3153 4.9902
##
## ---
## Signif. codes: 0 '***' 0.001 '**' 0.01 '*' 0.05 '.' 0.1 ' ' 1

bias.div.wood = rma.mv(yi,vi,mod = ~sqrt(vi), random = ~1|locale, data = div.wood.ef)
summary(bias.div.wood)

##
## Multivariate Meta-Analysis Model (k = 5; method: REML)
##
## logLik Deviance AIC BIC AICc
## -3.2480 6.4959 12.4959 9.7918 36.4959
##
## Variance Components:
##
## estim sqrt nlvls fixed factor
## sigma^2 0.2088 0.4570 3 no locale
##
## Test for Residual Heterogeneity:
## QE(df = 3) = 3.1150, p-val = 0.3742
##
## Test of Moderators (coefficient(s) 2):
## QM(df = 1) = 33.8164, p-val < .0001
##
## Model Results:
##
## estimate se zval pval ci.lb ci.ub
## intrcpt -2.0315 0.5483 -3.7053 0.0002 -3.1061 -0.9569 ***
## sqrt(vi) 5.3591 0.9216 5.8152 <.0001 3.5528 7.1653 ***
##
## ---
## Signif. codes: 0 '***' 0.001 '**' 0.01 '*' 0.05 '.' 0.1 ' ' 1

forest(test.div.wood,slab = div.wood.ef$author, annotate= FALSE, psize = 1.5, pch = 21,
 mlab = "Overall Mean Effect", addcred = TRUE, order = "obs")


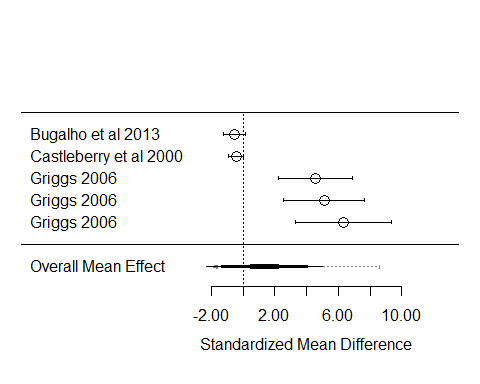


rs.div.wood = rstandard(test.div.wood)
hat.div.wood = hatvalues(test.div.wood)/mean(hatvalues(test.div.wood))
plot(hat.div.wood, rs.div.wood$resid, ylim = c(-6,6))
text(hat.div.wood, rs.div.wood$resid, labels = div.wood.ef$ID, cex= 1, pos = 2)
abline(h = -3)
abline(h = 3)
abline(v = 2)


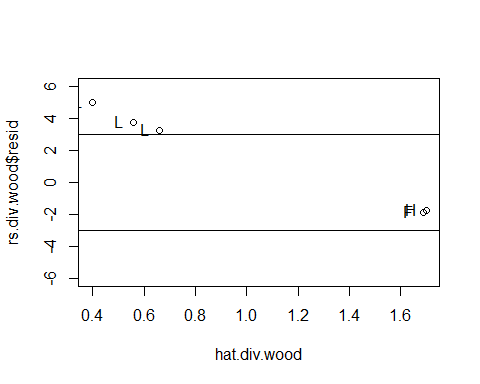


# ______________________________
div.herb.ef = escalc(measure = "SMD", m1i = t, m2i = c, sd1i = tv, sd2i = cv, n1i = tn, n2i = cn, data = div.herb)
test.div.herb = rma.mv(yi,vi,random = ~1|locale, data = div.herb.ef)
summary(test.div.herb)

##
## Multivariate Meta-Analysis Model (k = 7; method: REML)
##
## logLik Deviance AIC BIC AICc
## -25.0943 50.1887 54.1887 53.7722 58.1887
##
## Variance Components:
##
## estim sqrt nlvls fixed factor
## sigma^2 2.3512 1.5334 4 no locale
##
## Test for Heterogeneity:
## Q(df = 6) = 86.9911, p-val < .0001
##
## Model Results:
##
## estimate se zval pval ci.lb ci.ub
## -0.2708 0.8055 -0.3362 0.7367 -1.8494 1.3079
##
## ---
## Signif. codes: 0 '***' 0.001 '**' 0.01 '*' 0.05 '.' 0.1 ' ' 1

bias.div.herb = rma.mv(yi,vi,mod = ~sqrt(vi), random = ~1|locale, data = div.herb.ef)
summary(bias.div.herb)

##
## Multivariate Meta-Analysis Model (k = 7; method: REML)
##
## logLik Deviance AIC BIC AICc
## -21.3699 42.7397 48.7397 47.5681 72.7397
##
## Variance Components:
##
## estim sqrt nlvls fixed factor
## sigma^2 1.2512 1.1186 4 no locale
##
## Test for Residual Heterogeneity:
## QE(df = 5) = 71.2287, p-val < .0001
##
## Test of Moderators (coefficient(s) 2):
## QM(df = 1) = 5.4536, p-val = 0.0195
##
## Model Results:
##
## estimate se zval pval ci.lb ci.ub
## intrcpt -2.8199 1.2284 -2.2956 0.0217 -5.2275 -0.4123 *
## sqrt(vi) 4.8913 2.0945 2.3353 0.0195 0.7861 8.9965 *
##
## ---
## Signif. codes: 0 '***' 0.001 '**' 0.01 '*' 0.05 '.' 0.1 ' ' 1

forest(test.div.herb,slab = div.herb.ef$author, annotate= FALSE, psize = 1.5, pch = 21,
 mlab = "Overall Mean Effect", addcred = TRUE, order = "obs")


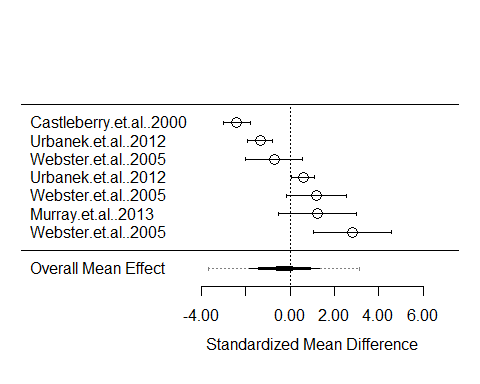


rs.div.herb = rstandard(test.div.herb)
hat.div.herb = hatvalues(test.div.herb)/mean(hatvalues(test.div.herb))
plot(hat.div.herb, rs.div.herb$resid, ylim = c(-6,6))
text(hat.div.herb, rs.div.herb$resid, labels = div.herb.ef$ID, cex= 1, pos = 2)
abline(h = -3)
abline(h = 3)
abline(v = 2)


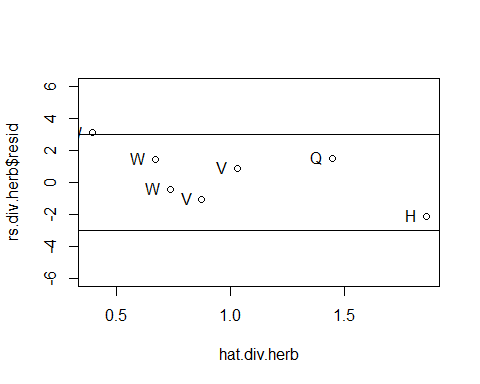

Supplement: Additional Information [file supp_plv119_plv119supp_file2.doc]
